# The influence of gender on the effects of aspirin in preventing myocardial infarction

**DOI:** 10.1186/1741-7015-5-29

**Published:** 2007-10-18

**Authors:** Todd Yerman, Wen Q Gan, Don D Sin

**Affiliations:** 1 Department of Medicine (Respiratory Division), University of British Columbia, and The James Hogg iCAPTURE Center for Cardiovascular and Pulmonary Research, St. Paul's Hospital, Vancouver, British Columbia, Canada

## Abstract

**Background:**

There is considerable variation in the effect of aspirin therapy reducing the risk of myocardial infarction (MI). Gender could be a potential explanatory factor for the variability. We conducted a systematic review and meta-analysis to determine whether gender mix might play a role in explaining the large variation of aspirin efficacy across primary and secondary MI prevention trials.

**Methods:**

Randomized placebo-controlled clinical trials that examined the efficacy of aspirin therapy on MI were identified by using the PUBMED database (1966 to October 2006). Weighted linear regression technique was used to determine the relationship between log-transformed relative risk (RR) of MI and the percentage of male participants in each trial. The reciprocal of the standard error of the RR in each trial (1/SE) was used as the weight.

**Results:**

A total of 23 trials (n = 113 494 participants) were identified. Overall, compared with placebo, aspirin reduced the risk of non-fatal MI (RR = 0.72, 95% confidence interval (CI) 0.64–0.81, p < 0.001) but not of fatal MI (RR = 0.88, 95% CI 0.75–1.03, p = 0.120). A total of 27% of the variation in the non-fatal MI results could be accounted for by considering the gender mix of the trials (p = 0.017). Trials that recruited predominantly men demonstrated the largest risk reduction in non-fatal MI (RR = 0.62, 95% CI 0.54–0.71), while trials that contained predominately women failed to demonstrate a significant risk reduction in non-fatal MI (RR = 0.87, 95% CI 0.71–1.06).

**Conclusion:**

Gender accounts for a substantial proportion of the variability in the efficacy of aspirin in reducing MI rates across these trials, and supports the notion that women might be less responsive to aspirin than men.

## Background

Although it is widely accepted that aspirin reduces the risk of myocardial infarction (MI) on average by 25%, there is considerable variation in the effect sizes reported across the trials ranging from zero to 50% relative to placebo [1]. To date, factors responsible for this heterogeneity have not been well studied. One potential explanatory variable is gender. There is emerging evidence to indicate that women have an increase risk of aspirin resistance compared to men, potentially making aspirin less effective in women [2]. Moreover, women who develop atherosclerosis tend to be older, have more co-morbid conditions and more extensive disease at the time of diagnosis, which might also interfere with the actions of aspirin [2]. Thus, the gender mix of published trials could be an important determinant of the variability in the reported findings. The primary purpose of this study was to determine the influence of gender mix on the reported efficacy of aspirin on fatal and non-fatal MI in published clinical trials.

## Methods

### Search for relevant studies

We conducted a comprehensive literature search by using the PUBMED electronic database (1966 to October 2006) to identify randomized placebo-controlled clinical trials that examined the efficacy of aspirin therapy on myocardial infarction (MI). We limited the search to randomized controlled trials conducted in human subjects and published in English language, using aspirin and MI-specific search terms. We supplemented the electronic search by probing the reference lists of retrieved articles and previous reviews on this topic, and by a search of the Antithrombotic Trialists' Collaboration website [3] and EMBASE. We also contacted primary authors where necessary for clarification of data.

### Study selection and data abstraction

The primary objective of this study was to determine the impact of gender mix on the reported efficacy of aspirin on MI rates. We excluded trials that: (1) had a follow-up period of less than 3 months; (2) co-administered aspirin with another agent; (3) prescribed aspirin for clinical indications other than for primary or secondary cardiovascular prevention (e.g. pain, headache, or arthritic symptoms); (4) did not have a placebo arm; (5) had a paucity of MI events (fewer than 10) during follow-up; or (6) had unacceptable methodological quality score (Jadad score of less than 3) [4]. From each retrieved article, two independent investigators abstracted the following information: project name, characteristics of participants, sample size, average age of the sample at baseline, proportionality of current smokers and male participants, duration of follow up, and dosage of aspirin (Table 1), and calculated the relative risks (RR) and 95% confidence intervals (CI) for fatal and non-fatal MI separately as well as combined [5-27]. Any questions or discrepancies regarding these data were resolved through iteration and consensus.

**Table 1:** Characteristics of selected trials

Author (year)	Trial name	Participants	Sample size	Mean age at baseline (year)	Current smoker (%)	Male (%)	Follow-up (year)	Aspirin dose (mg/day)
Ridker et al [26] (2005)	Women's Health Study (WHS)	Healthy women ≥ 45 years in USA	39 876	55	13	0	10.0	100†
de Gaetano [19] (2001)	Primary Prevention Project (PPP)	Patients with at least one of the major recognized cardiovascular risk factors in Italy	4 495	64	15	42	3.6	100
Cote et al [18] (1995)	Asymptomatic Cervical Bruit Study (ACBS)	Patients with asymptomatic carotid stenosis in Canada	372	67	37	45	2.3	325
ETDRS Study Group [13] (1992)	Early Treatment Diabetic Retinopathy Study report (ETDRS)	Patients with a clinical diagnosis of diabetes mellitus	3 711	18–70	44#	52	5.0	650
Juul-Moller et al [24] (1992)	Swedish Angina Pectoris Aspirin Trial (SAPAT)	Patients with chronic stable angina	2 035	67	16	52	4.2	75
Hansson et al [23] (1998)	Hypertension Optimal Treatment Study (HOT)	Patients with hypertension and diastolic blood pressure between 100 mmHg and 115 mmHg from countries in Europe, America, and Asia	18 790	62	16	53	3.8	75
Swedish cooperative [8] (1987)	Swedish Cooperative Study (Swedish Coop)	Patients with cerebral infarction, minor or major stroke	505	68	52	62	2.0	1 500
EAFT Study Group [14] (1993)	European Atrial Fibrillation Trial (EAFT)	Patients with non-rheumatic atrial fibrillation	782*	73	19	63	2.3	300
SALT Collaborative Group [11] (1991)	Swedish Aspirin Low-Dose Trial (SALT)	Patients after transient ischaemic attack (TIA) or minor stroke	1 360	67	25	66	2.7	75
Cairns et al [17] (1985)	Canadian multicenter trial (Canadian)	Patients with unstable angina who were hospitalized in coronary care units in Canada	278*	57	35	70	1.5	1 300
Stroke Prevention in Atrial Fibrillation Study group [12] (1991)	Stroke Prevention in Atrial Fibrillation Study (SPAF)	Patients with constant or intermittent atrial fibrillation	1 120*	67	16	71	1.3	325
Sorensen et al [27] (1983)	A Danish cooperative study (Danish Coop)	Patients experienced at least one reversible cerebral ischemic attack	203	59	24¶	73	2.0	1 000
Farrell et al [22] (1991)	United Kingdom transient ischaemic attack aspirin trial (UK-TIA)	Patients with a transient ischaemic attack or minor ischaemic stroke	2 435	60	53	73	4.0	300/1 200
Persantine-Aspirin Reinfarction Study Research Group [5] (1980)	Persantine-aspirin Reinfarction Study (PARIS)	Patients recovered from myocardial infarction	1 216*	56	27	77	3.4	972
Breddin et al [16] (1980)	German-Austrian aspirin trial (GAAT)	Patients who had survived a myocardial infarction for 30–42 days	626	45–70	58	78	2.0	1 500
Elwood and Sweetnam [21] (1979)	Aspirin and secondary mortality after myocardial infarction (Cardiff-II)	Patients with confirmed myocardial infarction	1 725‡	56	60	85	1.0	900
AMIS Study Group [6] (1980)	Aspirin myocardial infarction study (AMIS)	Patients experienced at least one myocardial infarction in USA	4 524	55	27	89	3.0	1 000
Elwood et al [20] (1974)	Secondary prevention of mortality from myocardial infarction (Cardiff-I)	Patients with recent myocardial infarction	1 239‡	55	NA	100	1.1	300
Coronary Drug Project Research Group [7] (1980)	Coronary Drug Project Aspirin Study (CDPA)	Patients with a history of myocardial infarction	1 529‡	NA	NA	100	1.8	324
Lewis et al [25] (1983)	Veterans Administration Cooperative Study (VACS)	Patients with unstable angina in USA	1 266	56	50	100	1.0	324
Steering Committee of the Physicians' Health Study Research Group [9] (1989)	Physicians' Health Study (PHS)	Healthy male physicians in USA	22 071	52	11	100	5.0	325†
The RISC Group [10] (1990)	Research Group on Instability in Coronary Artery Disease (RISC)	Men with unstable coronary artery disease in Southeast Sweden	796*	58	38	100	1.0	75
Medical Research Council's General Practice Research Framework Group [15] (1998)	Thrombosis prevention Trial (TPT)	Patients at high risk of cardiovascular disease in UK	2 540	58	41	100	4.0	75

NA: not available.

*Only the participants in placebo group and aspirin group were included.

† Every other day.

‡ Data are from [4].

¶ > 15 cigarettes/day.

# ≥ 6 cigarettes/day.

### Statistical analysis

We used both unweighted and weighted linear regression techniques to determine the relationship between log-transformed RR of MI and gender mix of the trial participants (i.e. the percentage of male or female subjects in each trial). In the weighted analysis, we used the reciprocal of the standard error of the RR in each trial (1/SE) as the weight. As the weighted and unweighted analysis produced similar results, we report on the weighted analysis only unless otherwise indicated. All tests were two-tailed in nature and were performed using SAS statistical software (version 9.1, SAS Institute, Carey, NC, USA).

In a separate analysis, we divided the original trials into tertile groups based on the percentage of male participants included in each trial. We used a random effects model to combine the results of the trials to take into account both within as well as between trial variances [28]. Data analyses were conducted using STATA statistical software (STATA release 9, STATA Corporation, College Station, Texas, USA) and Review Manager V. 4.2 (Revman The Cochrane Collaboration, Oxford, UK).

## Results

**Figure 1:**
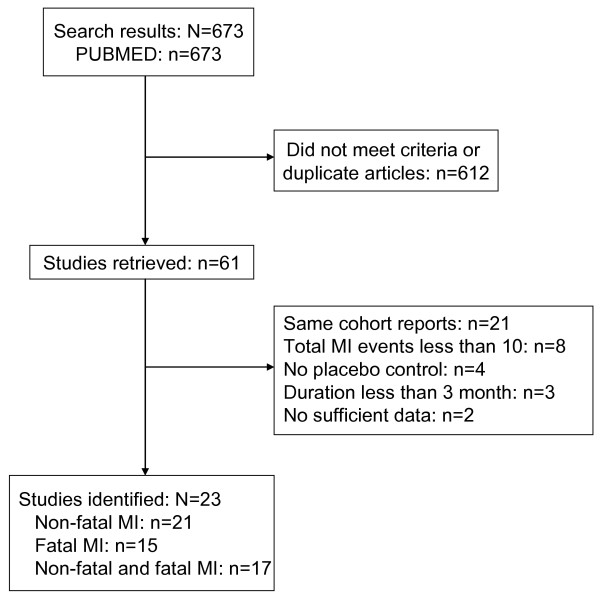
**Flow diagram of study selection**. MI, myocardial infarction.

The original search in PUBMED yielded 637 citations. EMBASE did not contribute any additional citations. The abstracts of these articles were selected and reviewed. Of these, 61 articles were retrieved for detailed examination. From these, 23 trials were selected as they met the inclusion and exclusion criteria of the study: 21 of these trials reported on non-fatal MI, 15 trials reported on fatal MI, and 17 reported on both fatal and non-fatal MI. The selection process is depicted in Figure 1. The baseline characteristics of participants in the selected trials are summarized in Table 1[5-27]. Overall, there were 113 494 participants in the 23 included trials; 49.3% were men. The baseline mean age of the trial participants ranged between 52 and 73 years; the prevalence of current smokers varied from 11% to 60%. The duration of follow-up was from 1–10 years and the dosage of aspirin varied from 75–1500 mg per day.

**Figure 2:**
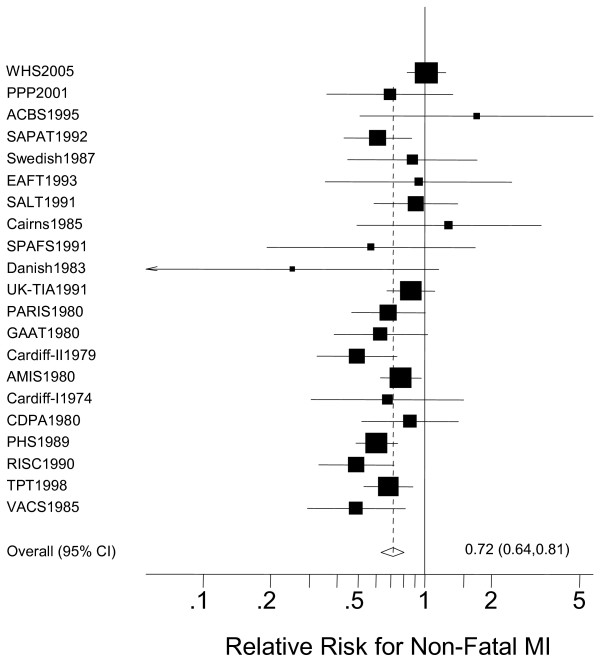
The effect of aspirin on the risk for non-fatal myocardial infarction (MI) compared with placebo.

The main findings of the study are summarized in Table 2. Overall, aspirin significantly reduced the risk of non-fatal MI compared with placebo (RR = 0.72, 95% CI 0.64–0.81, p < 0.001) (Figure 2). There was a trend towards lower fatal MI rates with aspirin (RR = 0.88, 95% CI 0.75–1.03, p = 0.120) (Table 2). Aspirin therapy significantly reduced the combined endpoint of fatal and non-fatal MI (RR = 0.79, 95% CI 0.72–0.87, p < 0.001) (Table 2).

**Table 2:** Relative risks and 95% confidence intervals of myocardial infarction in each trial

Source	Male (%)	Non–fatal MI	Fatal MI	Fatal and non-fatal MI
WHS [26] (2005)	0	1.02 (0.83–1.25)	1.17 (0.54–2.52)	1.03 (0.84–1.25)
PPP [19] (2001)	42	0.69 (0.36–1.34)	0.68 (0.19–2.40)	0.69 (0.39–1.23)
ACBS [18] (1995)	45	1.71 (0.51–5.75)	NA	NA
ETDRS [13] (1992)	52	NA	NA	0.85(0.73–1.00)
SAPAT [24] (1992)	52	0.61 (0.43–0.87)	1.02 (0.50–2.07)	0.68 (0.50–0.92)
HOT [23] (1998)	53	NA	NA	0.85 (0.69–1.05)
Swedish cooperative [8] (1987)	62	0.88 (0.45–1.72)	1.00 (0.33– 3.05)	0.91 (0.52, 1.60)
EAFT [14] (1993)	63	0.94 (0.35–2.47)	0.90 (0.52–1.56)	0.91 (0.56–1.46)
SALT [11] (1991)	66	0.91 (0.59–1.41)	0.65 (0.36–1.16)	0.80 (0.57–1.13)
Canadian group study [17] (1985)	70	1.29 (0.49–3.36)	0.54 (0.22–1.31)	0.80 (0.43–1.48)
SPAF [12] (1991)	71	0.57 (0.19–1.70)	NA	NA
Danish cooperative [27] (1983)	73	0.25 (0.05–1.16)	0.67 (0.20–2.31)	0.43 (0.17–1.08)
UK–TIA [22] (1991)	73	0.86 (0.68–1.11)	1.01 (0.74–1.39)	0.92 (0.77–1.11)
PARIS [5] (1980)	77	0.68 (0.47–1.01)	0.79 (0.55–1.15)	0.74 (0.57–0.95)
GAAT [16] (1980)	78	0.63 (0.39–1.03)	0.58 (0.30–1.12)	0.61 (0.42–0.89)
Cardiff–II [21] (1979)	85	0.49 (0.33–0.75)	NA	NA
AMIS [6] (1980)	89	0.78 (0.63–0.96)	1.08 (0.89–1.31)	0.93 (0.81–1.07)
Cardiff–I [20] (1974)	100	0.68 (0.31–1.49)	NA	NA
CDPA [7] (1980)	100	0.86 (0.52–1.42)	NA	NA
VACS [25] (1983)	100	0.49 (0.29–0.81)	0.17 (0.02–1.42)	0.45 (0.28–0.74)
PHS [9] (1989)	100	0.61 (0.49–0.75)	0.38 (0.19–0.80)	0.58 (0.47–0.72)
RISC [10] (1990)	100	0.49 (0.33–0.73)	NA	NA
TPT [15] (1998)	100	0.68 (0.53–0.89)	1.13 (0.78–1.63)	0.81 (0.66–0.99)
Total	49	0.72 (0.64–0.81)	0.88 (0.75–1.03)	0.79 (0.72–0.87)

MI, myocardial infarction; NA, not available.

**Figure 3:**
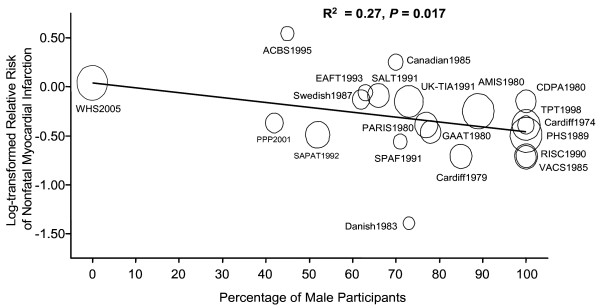
**The impact of gender mix on the reported efficacy of aspirin in reducing non-fatal myocardial infarction risk**. The regression line is weighted by the reciprocal of the standard error (1/SE) of the relative risk of each trial. The diameter of each circle is proportional to 1/SE of each trial.

In a meta-regression analysis, we observed a significant relationship between gender mix (i.e. proportionality of male or female participants in each trial) and the effectiveness of aspirin in reducing non-fatal MI rates in these trials (R^2 ^= 0.27, p = 0.017) (Figure 3). However, we failed to observe a significant impact of gender mix on the effect sizes for fatal MI (R^2 ^= 0.03, p = 0.514). The relationship between gender mix and the effectiveness of aspirin in reducing the combined end point of fatal and non-fatal MI was only marginally significant (R^2 ^= 0.21, p = 0.065). The inclusion of average age and smoking status of the trial participants made very little impact to the overall findings. For the end point of non-fatal MI, the R^2 ^value was 0.32 (p = 0.038). For fatal MI, it was 0.09 (p = 0.465) and for the combined end point of fatal and non-fatal MI, it was 0.28 (p = 0.072).

When the original trials were divided into tertile groups based on the gender mix (0–66%, 70–89%, 100% male participants in each trial), the beneficial effect of aspirin in reducing non-fatal MI was found to be the greatest in the tertile group containing the largest percentage of male participants (RR = 0.62, 95% CI 0.54–0.71), test for heterogeneity, p = 0.48). In contrast, the tertile group with the smallest percentage of male participants failed to demonstrate any benefits of aspirin in reducing non-fatal MI (RR = 0.87, 95% CI 0.71–1.06), test for heterogeneity, p = 0.26) (Table 3). Similar findings were observed in the analysis that combined non-fatal and fatal MI together (Table 3).

**Table 3:** Relative risks and 95% confidence intervals of myocardial infarction stratified by percentage of male participants

Male (%)	Non-fatal MI	p*	Fatal MI	p*	Both fatal and non-fatal MI	p*
0–66	0.87 (0.71–1.06)	0.26	0.87 (0.65–1.17)	0.86	0.86 (0.79–0.95)	0.52
70–89	0.72 (0.61–0.86)	0.23	0.91 (0.75–1.11)	0.24	0.82 (0.71–0.95)	0.13
100	0.62 (0.54–0.71)	0.48	0.55 (0.20–1.53)	0.01	0.63 (0.46–0.85)	0.02

Total	0.72 (0.64–0.81)	0.03	0.88 (0.75–1.03)	0.19	0.79 (0.72–0.87)	< 0.05

MI, myocardial infarction.

*Test for heterogeneity, p value is from χ^2 ^test. Ransom effects model was used in all combinations.

## Discussion

The findings of the present study indicate that aspirin is effective in reducing the risk for non-fatal MI. There was however considerable variation in the reported efficacy of aspirin across the trials. We found that approximately 27% of the total variation could be accounted for by considering the differences in the gender mix of the trials. In general, the trials that contained predominantly male subjects demonstrated large benefits of aspirin in reducing non-fatal MI rates. In contrast, trials that contained mostly female subjects failed to show any beneficial effect of aspirin on this end point. These data are consistent with the notion that aspirin therapy might be less effective in reducing non-fatal MI in women than in men.

Why aspirin would be less effective in reducing MI risk in women is largely a mystery. However, recent data indicate that women are more likely to demonstrate aspirin resistance compared to men. In a study by Chen and colleagues, women compared to men were 2.3 times more likely to be aspirin-resistant [29] and in the study by Gum and colleagues, women were 2.5 times more likely to demonstrate aspirin resistance [2]. The mechanisms underlying these observations are uncertain.

There are also emerging data demonstrating major structural and physiological differences in coronary vasculature between men and women [30]. For instance, women have smaller coronary vessels, which are generally stiffer than those in men owing to increased deposition of fibrotic tissue and remodeling of the vessel walls. Women are also more likely to demonstrate impaired vasodilatory responses to acetylcholine [31]. Moreover, when women develop atherosclerosis, their lesions are usually more diffuse and extensive than those observed in men [32]. Although in both men and women, the leading cause of morbidity and mortality is ischemic heart disease [33], women, especially in the younger age groups (less than 50 years of age), have short-term mortality rates that are twice those observed in men following MIs [34]. Our findings in the context of the emerging literature regarding possible aspirin resistance in women suggest that clinicians should be cautious in prescribing aspirin in women especially for primary prevention. Whether or not other anti-platelet agents would be more effective for women is unclear. Future clinical studies specifically powered to evaluate sex-specific end points will be needed to determine whether other anti-platelet agents might be more effective in women compared with aspirin.

There were limitations to the present study. Firstly, we did not have access to the primary data. As such, we could not determine the influence of other risk factors such as co-morbidities that have also been associated with aspirin-resistance [35]. These factors could further explain the large variation in results reported across the trials. Secondly, as with most systematic reviews, there was a possibility of publication or selection bias. To mitigate this possibility, we conducted an extensive search and included all trials that met the inclusion and exclusion criteria of the present study. Thirdly, we could not determine the mechanism by which gender modifies the efficacy of aspirin for MIs.

## Conclusion

The present study found that the gender mix of trials accounted for a substantial proportion of the variability in the reported efficacy of aspirin in reducing MI rates. Trials that recruited predominantly men demonstrated the largest benefits; while trials that recruited mostly women failed to demonstrate any benefits. These data are consistent with the notion that aspirin is less efficacious in women (for the reduction of MIs) and raise the possibility that women are more susceptible to aspirin resistance.

## Competing interests

This project is supported by ICEBERGS (Interdisciplinary Capacity Enhancement: Bridging Excellence in Respiratory Disease and Gender Studies), which is funded by the Canadian Institutes of Health Research (IGH/ICRH), the Canadian Lung Association, and the Heart and Stroke Foundation of Canada.

DDS is a Canada Research Chair in COPD and a senior scholar with the Michael Smith Foundation for Health Research.

## Authors' contributions

All of the authors made substantial contributions to the manuscript and had full access to the data. All authors read and approved the final version of the manuscript.

## Pre-publication history

The pre-publication history for this paper can be accessed here:


